# Book Reviews

**Published:** 1901-08

**Authors:** 


THE FEEDING OF INFANTS. Home Guide for Modifying Milk. By JOSEPH E. WINTERS, M. D., Professor of Diseases of Children, Cornell University Medical College. New York: E. P. Dutton & Co. 1901. [Price, 50 cents.]

Dr. Winter has written a little book with the subtitle, Home Guide for Modifying Milk, which perfectly describes its scope. Many physicians will be glad to place in the hands of mothers and nurses such a simple and practical guide. M. J. F.

